# Crystallization of TiO_2_-MoS_2_ Hybrid Material under Hydrothermal Treatment and Its Electrochemical Performance

**DOI:** 10.3390/ma13122706

**Published:** 2020-06-14

**Authors:** Katarzyna Siwińska-Ciesielczyk, Beata Kurc, Dominika Rymarowicz, Adam Kubiak, Adam Piasecki, Dariusz Moszyński, Teofil Jesionowski

**Affiliations:** 1 Institute of Chemical Technology and Engineering, Faculty of Chemical Technology, Poznan University of Technology, Berdychowo 4, PL-60965 Poznan, Poland; dominikarymarowicz@o2.pl (D.R.); adam.l.kubiak@doctorate.put.poznan.pl (A.K.); teofil.jesionowski@put.poznan.pl (T.J.); 2 Institute of Chemistry and Electrochemistry, Faculty of Chemical Technology, Poznan University of Technology, Berdychowo 4, PL-60965 Poznan, Poland; 3 Institute of Materials Science and Engineering, Faculty of Mechanical Engineering and Management, Poznan University of Technology, Jana Pawla II 24, PL-60965 Poznan, Poland; adam.piasecki@put.poznan.pl; 4 Institute of Inorganic Chemical Technology and Environment Engineering, Faculty of Chemical Technology and Engineering, West Pomeranian University of Technology, Szczecin, Piastów 42, PL-71065 Szczecin, Poland; dmoszynski@zut.edu.pl

**Keywords:** titanium dioxide, molybdenum disulfide, hydrothermal route, calcination, anode material

## Abstract

Hydrothermal crystallization was used to synthesize an advanced hybrid system containing titania and molybdenum disulfide (with a TiO_2_:MoS_2_ molar ratio of 1:1). The way in which the conditions of hydrothermal treatment (180 and 200 °C) and thermal treatment (500 °C) affect the physicochemical properties of the products was determined. A physicochemical analysis of the fabricated materials included the determination of the microstructure and morphology (scanning and transmission electron microscopy—SEM and TEM), crystalline structure (X-ray diffraction method—XRD), chemical surface composition (energy dispersive X-ray spectroscopy—EDS) and parameters of the porous structure (low-temperature N_2_ sorption), as well as the chemical surface concentration (X-ray photoelectron spectroscop—XPS). It is well known that lithium-ion batteries (LIBs) represent a renewable energy source and a type of energy storage device. The increased demand for energy means that new materials with higher energy and power densities continue to be the subject of investigation. The objective of this research was to obtain a new electrode (anode) component characterized by high work efficiency and good electrochemical properties. The synthesized TiO_2_-MoS_2_ material exhibited much better electrochemical stability than pure MoS_2_ (commercial), but with a specific capacity ca. 630 mAh/g at a current density of 100 mA/g.

## 1. Introduction

There are currently many scientific studies associated with the search for new materials and construction solutions enabling the further advancement of lithium-ion battery (LIB) technology. These batteries are considered to be one of the leading energy storage methods, and currently constitute a rapidly developing area of research [1,2,3]. The determination of optimal correlations between the electrode materials (cathode and anode) is of great importance in the construction of lithium-ion cells. These dependencies significantly influence the parameters of the cells, such as voltage, capacity, chemical stability, and the reversibility of charge/discharge reactions. The electrode materials should not only operate successfully with each other, but should also create a synergistic system together with the electrolyte and separator [4,5,6].

Graphene and its derivatives are the most widely used anode materials in commercial lithium-ion batteries [4,5,6,7,8]. The theoretical capacity of graphite is estimated to be 372 mAh/g. Structural deformation, initial loss of capacity and electrical disconnection are the chief disadvantages of the graphene electrode, causing limitations to its application [1,2,3]. For this reason, among the potential candidates to replace the commonly used carbon compounds as an anode material, titanium dioxide, molybdenum disulfide and hybrid materials based on them have been investigated [1,4,5,6,9].

Titanium dioxide and its derivatives have recently gained increasing popularity, especially as anode materials in lithium-ion batteries, since they enable the design of operating devices with high work safety [10,11,12]. Materials of this type offer relatively high surface area, high porosity, high chemical and thermal stability, a stable electrochemical window and increased cyclical efficiency [13,14,15,16]. However, many reports confirm that titanium dioxide has low electrical conductivity, which limits its use in electrochemical applications [17,18,19]. For this reason, many research centers worldwide are working to improve the electrochemical behavior of TiO_2_ and its derivatives. Such studies are mainly focused on increasing the conductivity and the electrical capacity of titanium dioxide structures by introducing selected compounds into its structure [9,10,11,19].

Among other materials, layered transition metal dichalcogenides, which have a structure similar to graphite, are used as promising active compounds for lithium storage [20,21,22,23]. Molybdenum disulfide is one of the most stable and versatile members of this family; due to its layered structure, it offers a large Brunauer-Emmett-Teller (BET) surface area, high charge carrier transport and high wear resistance [24,25]. Moreover, MoS_2_ has a high theoretical specific capacity (~670 mAh/g at a current density 1600 mA/g), which is three and a half times higher than the value for commercial graphite and permits easy lithium-ion charge/discharge [23,24]. On the other hand, MoS_2_ exhibits poor cycling stability and low rate capability, which limit its potential applications as an electrode material [26,27].

The most common strategy for improving the electrochemical performance of MoS_2_ is hybridization with TiO_2_, to achieve an improved rate capability and minimal degradation during cycling [9,23,28]. Many studies have demonstrated that to stabilize the structure of the hybrid material during the lithium insertion/extraction process, particles of TiO_2_ may be introduced as spacers between MoS_2_ nanosheets, thus making both components accessible to electrolytes [9,12,29,30]. Xu et al. [9] synthesized TiO_2_@MoS_2_ core-shell composites by a hydrothermal route in the presence of cetyltrimethylammonium bromide. The combination of these two active components in such composites leads to an electrode material with a high charge capacity of 871 mAh/g at a current density of 100 mA/g after 80 cycles, with a high Coulombic efficiency (CE) of 99.6%. Nano-TiO_2_-decorated MoS_2_ nanosheets were fabricated via a one-pot hydrothermal route by Zhu et al. [31]. Those authors reported that the synthesized materials exhibited excellent cycling stability and rate performance, delivering a capacity of 604 mAh/g after 100 cycles at a current density of 100 mA/g. Li et al. [32] synthesized a hierarchical nanocomposite of TiO_2_ nanowires decorated with molybdenum disulfide nanosheets (TiO_2_@MoS_2_) using a facile and low-cost glucose-assisted hydrothermal approach. The obtained nanocomposite exhibited a high initial discharge capacity of 862 mAh/g and a high initial CE of 84% at a current density of 100 mA/g. Moreover, the TiO_2_@MoS_2_ nanocomposite displayed an excellent rate capability, with a specific capacity of 414 mAh/g at 1000 mA/g. Zhu et al. [33] reported the effective synthesis of a three-dimensional (3D) flower-like MoS_2_/TiO_2_ nanohybrid via a two-step hydrothermal method. The obtained MoS_2_/TiO_2_ nanohybrid displayed excellent electrochemical performance with a high reversible capacity of 801 mAh/g at a current density of 100 mA/g after 50 cycles. In the work by Pang et al. [34], the authors fabricated a graphene@TiO_2_@MoS_2_ material, applying a multi-step chemical route. They reported that the graphene layer encapsulated onto TiO_2_@MoS_2_ microspheres facilitated charge transfer, together with an improvement in its electrochemical conductivity. The abovementioned method allowed the authors to obtain anode material in which mesoporous TiO_2_ reduces MoS_2_ aggregation and changes with the volume of active material. The graphene@TiO_2_@MoS_2_ hybrid material was characterized by a very high capacity of 980 mAh/g at a current density of 0.1 A/g, and a capacity retention of 89% after 200 cycles.

The energy crisis and environmental degradation have stimulated the rapid development of lithium-ion batteries and photocatalysts. TiO_2_-MoS_2_ hybrid materials have great potential and are still widely tested both in rechargeable lithium-ion batteries and in photocatalysis, due to their excellent properties. In addition to the high chemical stability of both MoS_2_ and TiO_2_, TiO_2_-MoS_2_ hybrids also have other advantages. These include the combination of the strong optical absorption of TiO_2_ with the high catalytic activity of MoS_2_, which is promising for photocatalysis, and the excellent structural stability of TiO_2_ together with the high theoretical specific capacity and unique layered structure of MoS_2_, because of which these composites are exciting prospective anode materials. The objective of the present research was to obtain a TiO_2_-MoS_2_ hybrid material, which would exhibit enhanced electrochemical performance as an anode material. To achieve this, we attempted to apply a hydrothermal method (with the use of different temperatures) for the synthesis of TiO_2_-MoS_2_ hybrid materials. The prepared hybrid systems were further treated at 500 °C to increase their crystallinity. Most importantly, we demonstrated that the calcination process can significantly improve the electrochemical performance of TiO_2_-MoS_2_ electrodes.

## 2. Materials and Methods

### 2.1. Materials

Titanium (IV) isopropoxide (TTIP, 97%), sodium molybdate dihydrate (Na_2_MoO_4_*2H_2_O, 99.5%), thiourea (CS(NH_2_)_2_, 99%), lithium hexafluorophosphate (LiPF_6_, 99.99%), ethylene carbonate (EC, 99%), dimethyl carbonate (DMC, 99%) and molybdenum disulfide (MoS_2_, 98%) were purchased from Sigma-Aldrich (Poland). Lithium foil (0.75 mm thick, 99.9%) was supplied by Sigma-Aldrich. Acetylene Black (AB, 99.9%), poly(vinylidene fluoride) (PVdF, M_W_ = 180,000) and *N*-methyl-2-pyrrolidinone (NMP, 99.5%) were purchased from Fluka. Deionized water was used in all experiments. All reagents were used without any further purification.

### 2.2. Fabrication of TiO_2_-MoS_2_ Hybrid Systems

TiO_2_-MoS_2_ hybrid systems (with a TiO_2_:MoS_2_ molar ratio of 1:1) were synthesized via a hydrothermal method. First, in a plastic vessel, an appropriate amount of inorganic precursor of Mo (sodium molybdate dehydrate) was dissolved in 50 cm^3^ of deionized water to form a transparent solution. Dissolution was carried out at room temperature in a closed vessel, using an IKAMAG R05 magnetic stirrer (IKA Werke GmbH, Staufen, Germany) at 500 rpm for 10 min. Then, an appropriate amount of the organic precursor of TiO_2_ (titanium(IV) isopropoxide) was added dropwise to the solution and stirred for 10 min to obtain a suspension. After that time, the organic precursor of S, thiourea (in an appropriate quantity), was added to the reaction mixture and stirred for 60 min to disperse it. The obtained solution was transferred to a Teflon-lined stainless steel autoclave, and hydrothermally treated at 180 or 200 °C for 24 h. The hydrothermal reactor was cooled at room temperature, and the obtained hybrids were filtered and washed three times with deionized water. The precipitates were then dried at 60 °C for 6 h. At the final stage, the samples were ground and sieved through an 80-μm sieve. Selected samples of the obtained hybrids were calcined at 500 °C (with heating rate 5 °C/min) for 4 h under inert gas (N_2_) using a Nabertherm P320 Controller (Lilienthal, Germany). The materials hydrothermally treated at 180 and 200 °C were labelled as TM_180 and TM_200, and those additionally calcined at 500 °C as TM_180_500 and TM_200_500.

### 2.3. Characterization of Synthesized Hybrid Materials

The physicochemical characterization of the fabricated materials included the determination of microstructure and morphology (SEM and TEM), crystalline structure (XRD), chemical surface composition (EDS), and the parameters of the porous structure (low-temperature N_2_ sorption). Additionally, to confirm the chemical surface concentration and the presence of the characteristic surface groups of the synthesized materials, X-ray photoelectron spectroscopy (XPS) and Fourier-transform infrared spectroscopy (FTIR) were used [35,36].

### 2.4. Electrochemical Performance

The TiO_2_-MoS_2_ hybrid material was used as a working electrode, lithium foil (Whatmann, 0.4–0.6 mm thick) as a counter, reference electrode, and separator, and 1 M of LiPF_6_ in EC/DMC—1:1 by volume (dissolved in a mixture of ethylene carbonate and dimethyl carbonate) as an electrolyte. The working electrodes were prepared by a slurry tape casting procedure. Typically, the mass of the electrode was as follows: Li: ca. 4.5 mg (0.785 cm^2^), TiO_2_-MoS_2_—3.5–4.0 mg. The slurry consisted of 70% wt. active hybrid materials, 15% wt. Acetylene Black and 15% wt. poly(vinylidene fluoride) (PVdF) dissolved in *N*-methyl-2-pyrrolidinone (NMP). The weight of the whole paste was 0.4 g. The slurry was tape-cast on the copper foil, and then the coated electrodes were dried at 120 °C for 24 h. Electrochemical tests were carried out in a Swagelok^®^ system. Galvanostatic charge/discharge tests were conducted on the battery measurement system at various current densities in the range 50–1000 mA/g with a cut-off voltage range of 0.01–3.0 V vs. Li/Li^+^ at room temperature. The impedance of cells (0.01 Hz and 100 kHz) and cyclic voltammetry (scan rate of 0.1 mV/s over a potential range of 0.01–3.0 V (vs. Li^+^/Li)) were determined using the GTM750 Potentiostat/Galvanostat/ZRA (Gamry Instruments, Warminster, USA).

## 3. Results and Discussion

### 3.1. Microstructure and Morphology

**Figure 1:**
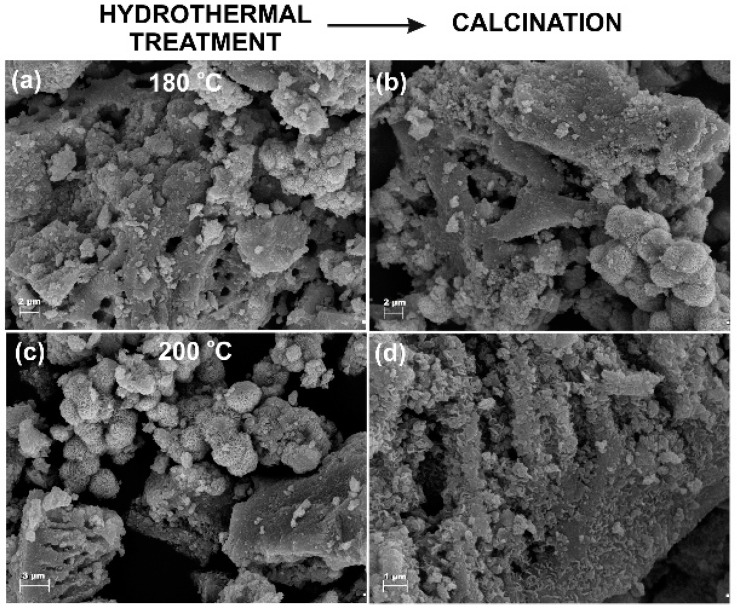
Scanning electron microscopy images of TiO_2_-MoS_2_ hybrid materials fabricated by a hydrothermal treatment at (**a**) 180 °C and (**c**) 200 °C, and the same materials additionally subjected to calcination at 500 °C (**b**,**d**).

For the visualization of the morphology and microstructure of the TiO_2_-MoS_2_ hybrid materials, scanning and transmission electron microscopy were applied. The SEM images of samples TM_180 and TM_180_500 indicate the presence of irregular and spherical shaped particles of TiO_2_, which show a high tendency to agglomerate, as well as flower-shaped MoS_2_ particles (Figure 1a,b). The SEM image of sample TM_200 (TiO_2_-MoS_2_ hybrid system hydrothermally treated at 200 °C; Figure 1c) shows the presence of numerous flower-shaped MoS_2_ particles, which probably covered the irregular particles of TiO_2_. The SEM image for a sample hydrothermally treated at 200 °C and additionally calcined at 500 °C (TM_200_500; Figure 1d) indicates irregular shaped particles and nanoplates merging into larger clusters. Numerous macropores are also visible.

**Figure 2:**
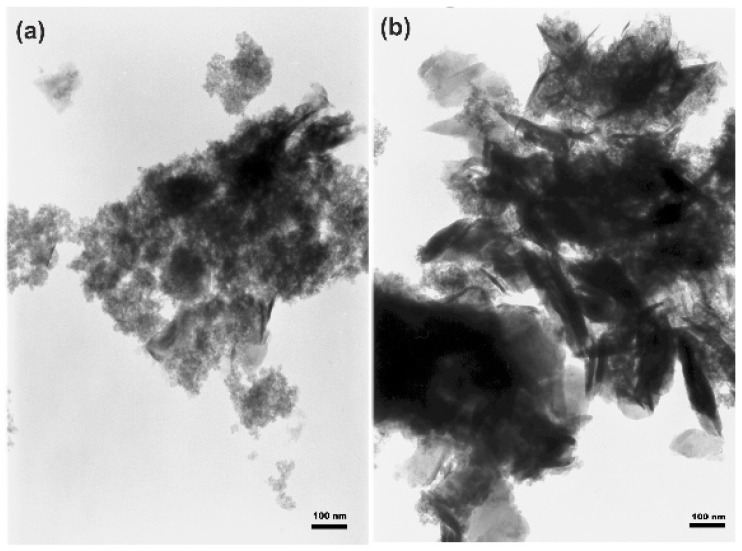
Transmission electron microscopy images of TiO_2_-MoS_2_ hybrid materials obtained via a hydrothermal method at (**a**) 180 °C and (**b**) 200 °C.

The TEM images for TiO_2_-MoS_2_ samples hydrothermally treated at 180 and 200 °C (Figure 2a,b) indicate the presence of spherical particles characteristic of titanium dioxide, as well as sheets, which can be attributed to the presence of molybdenum disulfide.

### 3.2. Crystalline Structure

**Figure 3:**
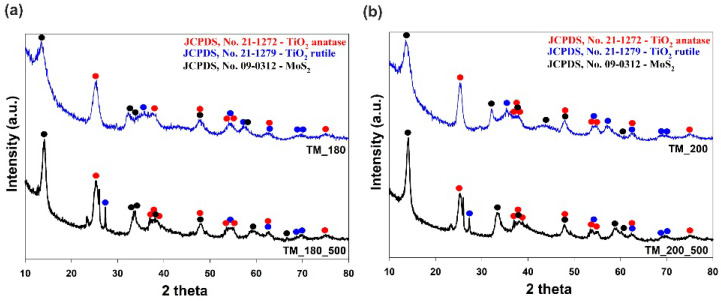
Wide-angle X-ray diffraction (WAXS) patterns of TiO_2_-MoS_2_ hybrid materials obtained via a hydrothermal method at (**a**) 180 °C and (**b**) 200 °C, and their calcined derivatives.

The determination of the crystalline structure of the synthesized TiO_2_-MoS_2_ hybrid materials was a key goal of the physicochemical analysis. The XRD results are shown in Figure 3.

The XRD pattern of sample TM_180 (TiO_2_-MoS_2_ hybrid system hydrothermally treated at 180 °C; Figure 3a) indicates the presence of reflections at 2θ values of 25.28, 36.95, 37.80, 38.88, 48.05, 53.89, 55.06, 62.69 and 75.03°, which are strictly related to the anatase phase space group: I4_1_/amd no. 141 (JCPDS Files No. 21-1272). Moreover, for the analyzed sample, the diffraction peaks recorded at 2θ = 14.33, 32.93, 33.96, 38.15, 47.96 and 60.31° (JCPDS Files No. 09-0312) confirmed the formation of the MoS_2_ phase space group P6_3_/mmc no. 194. Moreover, it should be noted that the intensities of MoS_2_ reflections are much weaker than those of TiO_2_, demonstrating the poor crystallinity of the MoS_2_ phase [37]. The interpretation of the XRD pattern of a TiO_2_-MoS_2_ hybrid system hydrothermally treated at 200 °C (Figure 3b) demonstrates that increasing the temperature of the thermal treatment leads to a product with slightly better crystallinity. Powder diffraction patterns for TiO_2_-MoS_2_ hybrid systems hydrothermally treated at 180 and 200 °C and additionally calcined at 500 °C (Figure 3a,b; black curves) indicate that the calcination process leads to products in which the reflections from MoS_2_ are higher. It was confirmed that the higher temperature of thermal treatment led to final products with more intensive reflections characteristic of the MoS_2_ structure. Xu et al. [9] also observed that, after annealing, the reflection characteristic of MoS_2_ at 2θ = 14.33° is sharper, which suggests the better crystallinity of TiO_2_@MoS_2_ composites after annealing. For the analyzed samples, on the XRD patterns (Figure 3a,b), a reflection at 2θ = 27.45° characteristic of rutile space group P4_2_/mnm no. 136 (JCPDS No. 21-1279) was observed. Moreover, XRD patterns for TiO_2_-MoS_2_ hybrid systems hydrothermally treated at 180 and 200 °C and additionally calcined at 500 °C (Figure 3a,b) indicated the presence of reflections at 2θ = 23.31, 25.67 and 27.26°, probably related to the crystallization of the orthorhombic phase γ-MoO_3_ (JCPDS No. 05-0508) [38]. Prabhakar Vattikuti et al. [38] also reported the formation of the orthorhombic phase γ-MoO_3_ space group P6_3_/mmc no. 194, which is probably related to the oxidization of some of the MoS_2_ in the hydrothermal process. Moreover, Li et al. [32] demonstrated that the hydrothermal route is an effective method enabling the successful coating of MoS_2_ sheets in a TiO_2_@MoS_2_ nanocomposite. They noted that, after thermal treatment of the nanocomposite at 800 °C, most of the reflections became more intense and sharper, while the intensities of the diffraction peaks of TiO_2_ decreased. Moreover, for fabricated hybrid materials, the phase composition (% wt.) and the lattice parameter of each phase were determined from the Rietveld method using the Fullprof software [39]. The results are presented in Table 1.

### 3.3. Surface Chemical Composition

**Figure 4:**
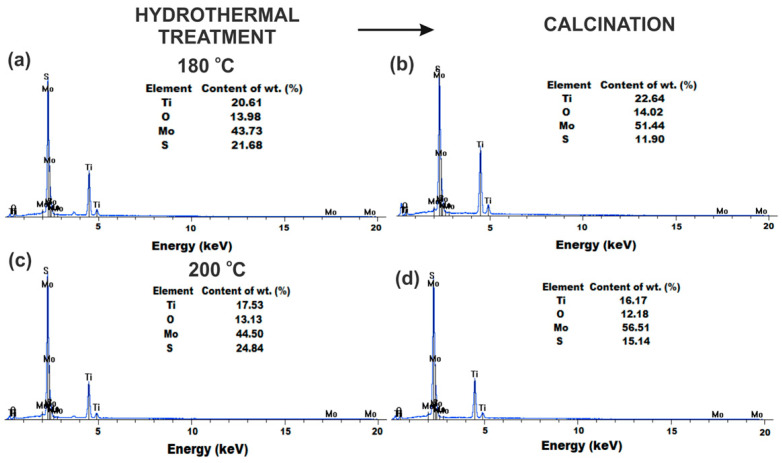
EDS spectra of TiO_2_-MoS_2_ hybrid materials obtained via a hydrothermal method at (**a**) 180 °C and (**c**) 200 °C, and the same materials additionally subjected to calcination at 500 °C (**b**,**d**).

To investigate the surface elemental composition of the synthesized TiO_2_-MoS_2_ hybrid systems, energy-dispersive X-ray spectrometry (EDS) was used (Figure 4).

The results indicate that the TiO_2_-MoS_2_ hybrid system hydrothermally treated at 200 °C and additionally calcined at 500 °C (TM_200_500; Figure 4d) had the highest content of molybdenum (56.51%) and the lowest content of titanium (16.17%) among all of the synthesized materials. Furthermore, the results of the EDS analysis for all samples show that the percentage content of titanium decreased, and that of molybdenum increased, when the temperature of the hydrothermal treatment increased from 180 to 200 °C. The EDS results confirmed the effectiveness of the proposed hydrothermal method in the synthesis of TiO_2_-MoS_2_ hybrid materials. Moreover, it was demonstrated that the temperature of hydrothermal treatment has a small effect on the surface composition of the analyzed materials. It was shown that the hydrothermal method makes it possible to obtain hybrid materials with strictly defined properties.

### 3.4. Textural Properties

**Figure 5:**
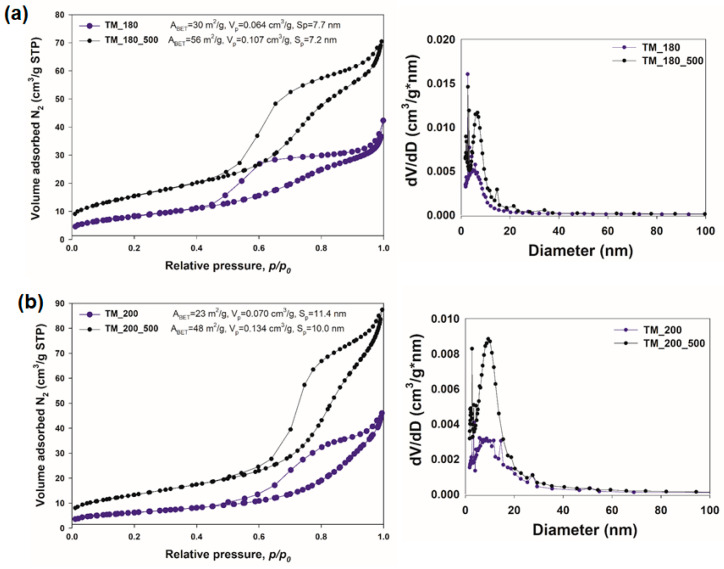
N_2_ adsorption/desorption isotherms and pore size distribution of TiO_2_-MoS_2_ hybrid materials obtained via a hydrothermal method at (**a**) 180 °C and (**b**) 200 °C and additionally subjected to calcination.

Many studies have proven that the surface area of an electrode material affects the efficiency of the electrochemical process [40,41]. In view of this fact, to investigate the textural properties of the synthesized hybrid products, low-temperature N_2_ sorption was carried out (Figure 5). The pore size distribution (S_p_) and pore volume (V_p_) were analyzed using the Barrett–Joyner–Halenda (BJH) method, and the surface area (A_BET_) was calculated using the Brunauer–Emmett–Teller (BET) method. The isotherm curves of the TiO_2_-MoS_2_ hybrid materials (Figure 5) are in good agreement with type IV isotherms with H3 hysteresis behavior, which are characteristic of mesoporous products according to the International Union of Pure and Applied Chemistry (IUPAC) classification [40].

The values obtained for parameters of the porous structure showed that TiO_2_-MoS_2_ hybrid systems hydrothermally treated at 180 and 200 °C without calcination (samples TM_180 and TM_200; Figure 5a,b) had a lower BET surface area than the corresponding products which were additionally calcined at 500 °C. The BET surface area was 30 m^2^/g and 23 m^2^/g for samples TM_180 and TM_200, respectively. The lower value of the BET surface area for sample TM_200 confirms that the higher temperature of the hydrothermal treatment causes greater sintering of the particles of the material and the formation of agglomerates, which is accompanied by the collapse of the porous structure of the material. A high temperature of hydrothermal treatment accelerates the evaporation of water from the fabricated hybrid material, contributing to the loss of surface hydroxyl (-OH) groups, whose presence increases the surface area [42]. The mean pore diameter of these materials was 7.7 nm (sample TM_180) and 11.4 nm (sample TM_200), and the total pore volume was equal to 0.064 cm^3^/g and 0.070 cm^3^/g for samples hydrothermally treated at 180 and 200 °C, respectively. After the calcination of the materials at 500 °C, the resultant BET surface areas were found to be 56 m^2^/g and 48 m^2^/g for samples TM_180_500 and TM_200_500, respectively. The total pore volume of sample TM_180_500 was 0.107 cm^3^/g, and the mean pore diameter 7.2 nm, while the mean pore diameter of sample TM_200_500 was 10.0 nm and the total pore volume 0.134 cm^3^/g.

An analysis of the parameters of the porous structure indicated that thermal treatment at 500 °C positively affected the value of the BET surface area of the fabricated hybrid materials. The higher BET surface area of TiO_2_-MoS_2_ materials is probably related to the formation of the MoO_3_ crystalline phase, which is confirmed by the XRD analysis. Moreover, N_2_ adsorption/desorption isotherms for all synthesized materials (Figure 5) exhibited a significant opening of the hysteresis loops, which indicates a developed mesoporous structure. The presence of mesopores in the synthesized hybrid materials may be desirable for energy storage applications.

### 3.5. FTIR Analysis

**Figure 6:**
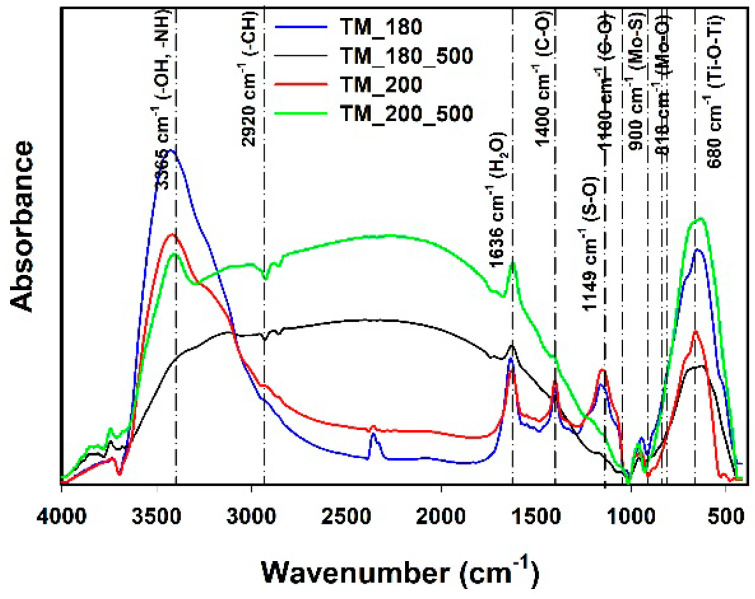
FTIR spectra of TiO_2_-MoS_2_ hybrid materials synthesized by a hydrothermal method at different temperatures, and their calcined derivatives.

Fourier transform infrared spectroscopy was used to identify changes in the chemical structure of the synthesized hybrid systems based on TiO_2_ and MoS_2_. The FTIR spectra of the TiO_2_-MoS_2_ materials are shown in Figure 6.

The FTIR analysis for the obtained hybrid systems showed the presence of an absorption band at wavenumber 680 cm^−1^, corresponding to stretching vibrations of the Ti-O-Ti group [43,44]. Symmetric and asymmetric stretching vibrations of the Mo-O group at 818 cm^−1^, 773 cm^−1^ and 675 cm^−1^ were also observed [45]. Moreover, the absorption band present at wavenumber 900 cm^−1^ indicates the presence of Mo-S bonds [43]. The bands at the wavenumbers 1100 cm^−1^ and 1400 cm^−1^ may correspond to stretching vibrations of the C-O group, while the signal at wavenumber 1500 cm^−1^ may indicate the presence of stretching vibrations of the N-H group [42]. The bands at 1636 cm^−1^ and 3365 cm^−1^ are attributed to hydroxyl groups (-OH) and water on MoS_2_ [46]. The band located at 1149 cm^−1^ corresponds to asymmetric S=O and S-O stretching vibrations [46].

The interpretation of the results indicates the presence of Ti-O-Ti, Mo-S, and -OH groups in the synthesized hybrid materials. It was shown that a change in the temperature of hydrothermal treatment (from 180 to 200 °C) does not affect the intensity of the characteristic bands. On the other hand, calcination caused a decrease in band intensity for the -OH group, although it did not change the intensity of the characteristic bands from TiO_2_.

### 3.6. XPS Analysis

**Figure 7:**
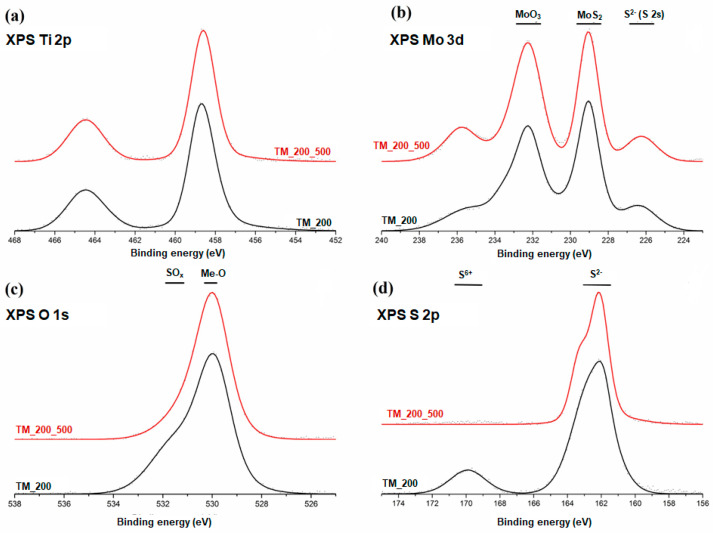
X-ray photoelectron spectra of TM_200 and TM_200_500 hybrid materials: (**a**) Ti 2p line, (**b**) Mo 3d line, (**c**) O 1s line and (**d**) S 2p line.

Detailed high-resolution X-ray photoelectron spectra were acquired for the fresh sample TM_200 and for the corresponding sample after calcination, TM_200_500. They are presented in Figure 7. The XPS Ti 2p spectrum observed for both analyzed samples consists of a doublet of peaks originating from a spin–orbit splitting of Ti 2p orbitals (see Figure 7a). The most intense peak, the component Ti 2p_3/2_, has a maximum at a binding energy of 458.8 eV. The shift of the Ti 2p_1/2_ component is 5.7 eV. Both the position and the shift are characteristic of TiO_2_ [47,48]. The position and the envelope of the spectra recorded for samples before and after calcination are virtually identical, which indicates a lack of chemical transformation of titania during this process.

Some variations of the peak envelopes are observed after the calcination of sample TM_200 (TM_200_500) in binding energy regions characteristic of molybdenum, oxygen and sulfur (see Figure 7b–d). The X-ray photoelectron spectrum of the Mo 3d region is complex and consists of several local maxima. At approximately 226 eV, a component originating from sulfur atoms (XPS S 2s peak) is observed [49]. The main maximum at 229.0 eV originates from the electrons of the Mo 3d_5/2_ component [50]. This position is attributed to the presence of MoS_2_. Mo 3d orbitals have a spin-orbit splitting of about 3.2 eV. Therefore, a Mo 3d_3/2_ component coming from molybdenum atoms bound with sulfur atoms should be located at a binding energy of approximately 230 eV. There is a prominent peak at this position. However, it is a superposition of two components: Mo 3d_3/2_ from MoS_2_, and another Mo 3d_5/2_ component originating from the electrons of Mo^6+^ ions in MoO_3_ [51]. This last component is confirmed by the presence of its spin–orbit component (a local maximum at approximately 335.6 eV). The observed Mo 3d spectrum envelopes indicate that the surface of the sample consists of MoS_2_ as well as MoO_3_, before and after calcination. However, after calcination, the local maximum at 335.6 eV, characteristic of MoO_3_, is more prominent. In spite of the inert atmosphere in which the calcination at 500 °C was carried out, some oxidation of molybdenum compounds takes place during this process.

An analysis of the X-ray photoelectron spectrum for oxygen atoms (XPS O 1s; Figure 7c) confirms the presence of the transition metal oxides, since its maximum is located at a characteristic region around 530 eV [48,52]. TiO_2_ and MoO_3_ are indistinguishable in this analysis. The XPS S 2p (Figure 7d) spectrum acquired for the TM_200 sample consists of two maxima. The more intense of these is located at a binding energy of approximately 162 eV and corresponds to the presence of MoS_2_ [49]. The other local maximum, centered at a binding energy of 169.6 eV, is usually attributed to the presence of hexavalent sulfur atoms, S^6+^, as in sulfates [51]. The presence of the latter is corroborated by a notable shoulder in the XPS O 1s spectrum at a binding energy of approximately 532 eV, which can also be attributed to SO_4_^2-^ ions [51]. After calcination, both components originating from S-O interactions disappear, indicating that the elevated temperature induced the decomposition of these bonds.

The results described above indicate that the surface of the TiO_2_-MoS_2_ hybrid is a complex structure. The predominant compounds are titanium dioxide and molybdenum disulfide, as was also observed with the use of other analytical methods. However, the substantial oxidation of MoS_2_ to MoO_3_ is observed, and this is more prominent for the calcined sample. The surface of the fresh sample also contains SO_4_^2-^ ions, which are removed at an elevated temperature.

### 3.7. Electrochemical Performance

It has been noted in recent years that, just as graphene is obtained from graphite, layers of individual atoms can be obtained from many other crystals. Such layers have been produced for, among others, transition metal chalcogenides such as sulfides, selenides and tellurides. Molybdenum disulfide (MoS_2_) layers have proved to be a particularly interesting material. This compound occurs in nature as molybdenite, a crystalline mineral often taking the form of characteristic hexagonal plates with a silvery color. Molybdenite, which resembles graphite and has often been confused with it, is found in rocks around the world. It has been used for many years in the production of lubricants and metal alloys. As with graphite, the properties of monatomic MoS_2_ layers have long gone unnoticed.

From the point of view of applications in electronics, layered molybdenum disulfide has a significant advantage over graphene: it exhibits what is called an energy gap. The existence of this gap means that electrons cannot absorb any energy, and, by applying an electric field, the material can be switched between a state in which it conducts a current and a state in which it behaves like an insulator.

Scientific research related to the search for new material and construction solutions, enabling the further progress of lithium-ion (LIB) technology, which is considered one of the leading energy storage methods, is currently a dynamically developing scientific and research trend. TiO_2_-MoS_2_ has become very popular in various applications related to the environment and energy, as evidenced by numerous scientific publications on this type of material in recent years. Several recent review articles have focused on the synthesis and application of hybrid materials based on TiO_2_ [53,54,55,56,57,58,59,60,61,62] and MoS_2_ [63,64,65,66,67,68,69,70,71,72,73,74].

Wang et al. [27] described the synthesis of MoS_2_-based composites quite extensively—he included broad applications for electrochemical energy storage, including LIB, sodium ion batteries (SIB) and supercapacitors. Additionally, the application of a wide range of MoS_2_ and TiO_2_ based materials in electro- and photocatalysis, solar cells and supercapacitors, electronic devices, sensors, bioapplications, LIB and SIB was discussed. On the other hand, Tian et al. [75] analyzed TiO_2_-based heterostructures for a wide range of applications: dye-sensitive solar cells, sensors, LIB, biomedicine, for photocatalysis, catalysis and lithium-ion cells. Moreover, titania is commonly used in solar cells, gas sensors, photonic crystals and self-cleaning coatings. Its universality results from its chemical stability, environmental friendliness and low cost. Titanium dioxide’s behavior in the abovementioned applications (especially for solar cells) depends on its crystallinity, crystalline phase, surface area and morphology [76].

It is worth noting that these materials are characterized with high chemical and thermal stability. An additional advantage is their biocompatibility and relatively large surface area (associated with well-developed porosity). Their stable electrochemical window and increased cyclic efficiency is of great significance when they are applied in battery fabrication. All these features mean that systems based on titanium dioxide can be the active components of anode materials in lithium-ion batteries.

**Figure 8:**
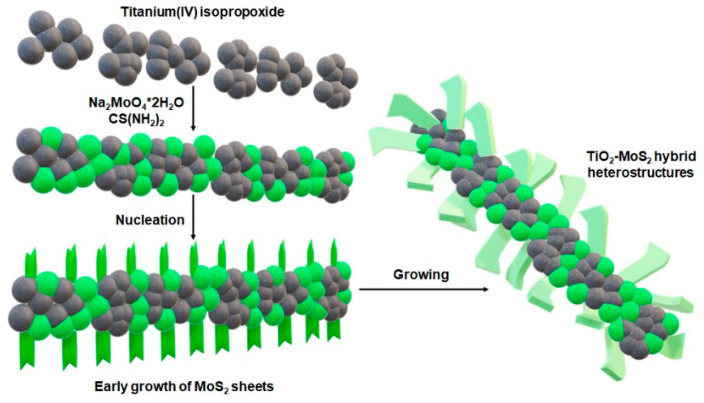
Schematic diagram of the nucleation and growth of MoS_2_ sheets on TiO_2_ particles.

It should be mentioned that hydrothermal and solvothermal methods are suitable for the preparation of MoS_2_ nanocomponents using Mo and S ions. The TiO_2_ matrix shows excellent chemical stability under these conditions. Therefore, hydrothermal and solvothermal methods are most often used for the synthesis of MoS_2_ on the TiO_2_ surface. Figure 8 illustrates the method in which two stages are marked: nucleation and growth.

The nucleation stage is very important. TiO_2_ introduced in the reaction solution acts as a substrate or matrix for MoS_2_ nucleation, which is called heterogeneous nucleation [77]. The surface structure (crystal lattice and surface) of the TiO_2_ matrix has been shown to have a strong effect on the heterogeneous MoS_2_ nucleation on this matrix. After successful nucleation, MoS_2_ is present on the TiO_2_ matrix. This has a significant impact on the size and density of MoS_2_ nanoparticles, they are easily controlled by adjusting the reaction parameters: growth time, growth temperature, initial reagent concentration, pH value and additives.

Titanium dioxide samples with a single anatase phase using 0.5 M NaNO_3_:0.5 M KNO_3_ (TiO_2_-I) and 0.88 M LiNO_3_:0.12 M LiCl (TiO_2_-II) salts were fabricated by Reddy et al. [78]. The cyclic voltammetry studies for prepared materials identified characteristic cathodic and anodic redox peaks at ∼1.7 and ∼2.0 V vs. Li/Li^+^, in the voltage range 1.0–2.8 V, respectively. The results of the galvanostatic cycling tests for the TiO_2_-I sample showed the first discharge/charge capacity values at 244 and 198 mAh/g for at a current of 33 mA/g. On the other hand, the TiO_2_-II product was characterized with less capacity fade during cycling and delivered first discharge/charge capacity values at 340 and 253 mAh/g.

Petnikota and co-authors [79] proposed the simple solid state ‘Graphenothermal Reduction’ method for the synthesis of exfoliated graphene oxide (EG)/MoO_2_ composites (with 46% wt. of EG). The fabricated EG/MoO_2_ composite was tested as an anode material. The tested anode material was characterized with reversible capacity values of about 878 and 431 mAh/g at current densities of 100 and 1000 mA/g after 100 cycles. Moreover, the exfoliated graphene oxide (EG)/MoO_2_ composite exhibited stable cycling for up to 100 cycles at 1000 mA/g with a capacity retention of ~100%. Electrochemical and structural studies indicated that lithium’s intercalation into the MoO_2_ structure was transformed into conversion reactions. This fact had an ideal effect on increasing the capacity of the tested materials at a lower current density. Moreover, due to differences in reaction kinetics and Li diffusion coefficients, the intercalation mechanism at a higher current is favorable for the entire cycle [80].

**Figure 9:**
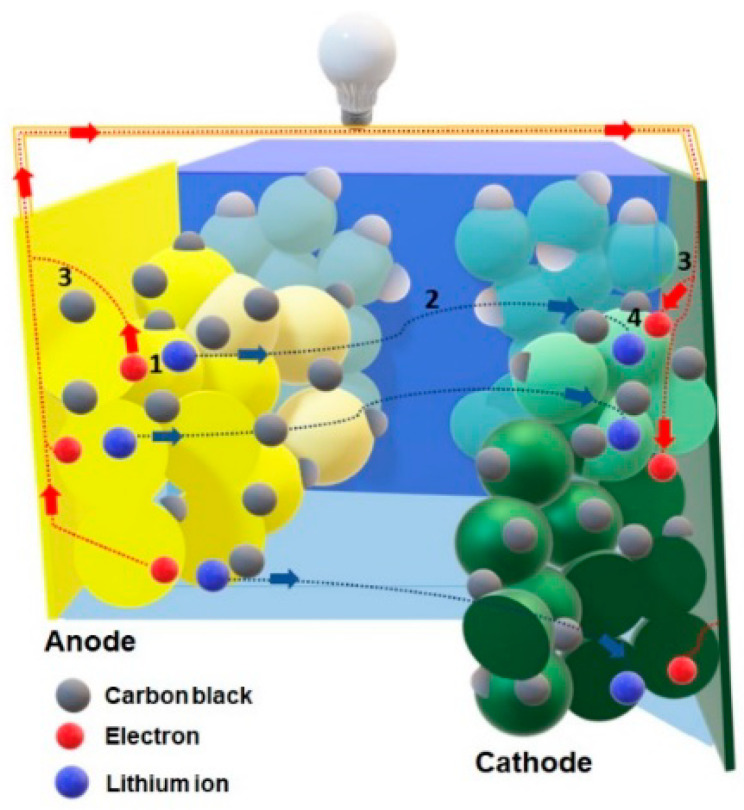
Operation of a lithium-ion cell.

Zhou et al. [81], for the first time, synthesized a nano-TiO_2_ composite coated with MoS_2_ particles by the hydrothermal method. Using Na_2_MoO_4_*2H_2_O as the Mo source and C_2_H_5_N_S_ as the S source, they were able to make a large number of MoS_2_-TiO_2_-based composites (200 °C for 24 h). Studies on the controlled synthesis of MoS_2_-coated TiO_2_ composites with various morphologies are popular [31,81,82,83,84,85,86]. MoS_2_-TiO_2_-based composites were synthesized by adjusting controlled factors. TiO_2_ surface states directly affect nucleation and growth. The hydrothermal controlled synthesis of MoS_2_-TiO_2_-based composites is determined by the surface states of TiO_2_ and the reaction parameters. All this also has a significant impact on the electrochemical properties of the resulting system. The proposed reaction mechanism is shown in Figure 9.

(1) Li-ion and electrons travel in reverse directions by diffusion in the solid and in parallel, disconnecting from the anode material; (2) the Li-ion shifts to the electrode/electrolyte boundary and passes through the electrolyte; (3) an electron driven by a higher potential from the cathode side flows through the anode particles and goes to the current collector instead of entering the electrolyte, then migrates through the external circuit to power the device; (4) the electron and lithium ion are simultaneously introduced into the cathode materials by semiconductor diffusion.

Transition metal sulfides do not exhibit such high electronic conductivity. Thus, a high rate of electron transfer from the current collector to an electroactive material was possible thanks to the synthesis of transition metal III–IV group carbon/nitrogen (M_n+1_AX_n_/MAX) phase materials. The various solid-state compounds applied to the fabrication of those types of materials are very often characterized with much higher electrochemical conductivity than that which is conventionally used carbonaceous materials. The abovementioned materials are very often used as surface modification agents. In the work by Ivanishcheva et al. [87], the authors proposed the using of titanium carbosilicide (Ti_3_SiC_2_) to improve the performance of lithium transition metal phosphate cathode materials, such as Li_3_V_2_(PO_4_)_3_/C or LiFePO_4_/C. It should be mentioned that MAX compounds do not participate in lithium ion transport, but intensify the motion of electrons within active material and thus Li-ion intercalation. Moreover, this compound is very attractive because of its function as protective layer between the electrode-electrolyte interface, which allows for a higher stability after cycling. However, the discharge capacity values for Ti_3_SiC_2_ samples were still not high and equal to 95 mAh/g, 88 mAh/g after 100 cycles at current 1C for Li_3_V_2_(PO_4_)_3_/C and LiFePO_4_/C cathode materials, respectively.

Moreover, it should be mentioned that the poor conductivity of some materials can be improved by using carbonaceous materials properly located in the intermolecular spaces such as carbon shells [88]. Ren with co-authors [88], proposed the sol-gel method assisted by hydrothermal treatment to fabricate the core-shell Li_3_V_2_(PO_4_)_3_@C composite as a cathode material for LIBs. Electrochemical tests indicated that carbon shells incorporated onto Li_3_V_2_(PO_4_)_3_ material improved the diffusion process of lithium ions and electrical conductivity. The discharge capacity of the fabricated Li_3_V_2_(PO_4_)_3_@C material was 125.9 mAh/g at a current density of 28 mA/g after 50 cycles. This value was two times higher than pure Li_3_V_2_(PO_4_)_3_—68.1 mAh/g after 30 cycles at the same current density. Moreover, the retention rate of the cathode material modified by the carbon shell reached almost 98.5%. This fact is caused by the naturally high adsorptive properties and BET surface area of carbon-based materials, because they play a considerable role in their electrochemical applications.

**Figure 10:**
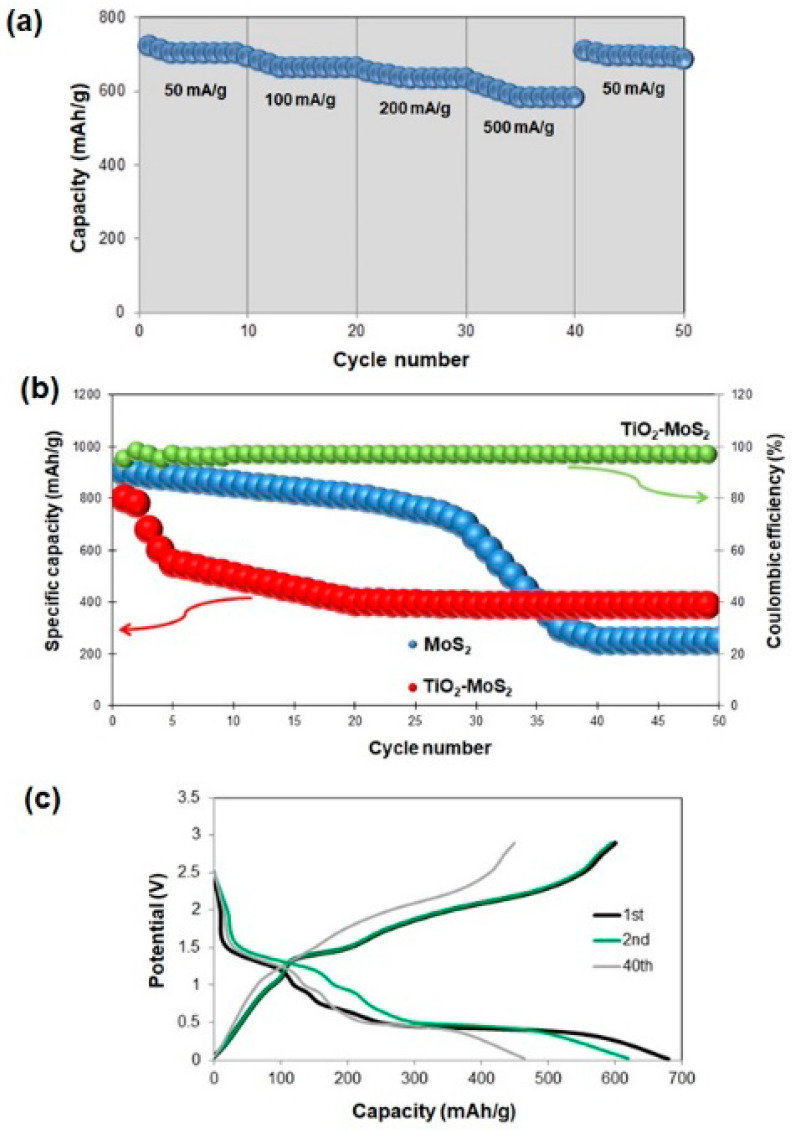
(**a**) Rate behavior of the TiO_2_-MoS_2_ hybrid material at different current densities, (**b**) cycling performance of the TiO_2_-MoS_2_ hybrid and MoS_2_ electrodes, and Coulombic efficiency of the TiO_2_-MoS_2_ material at a current density of 100 mA/g, (**c**) charge/discharge voltage profiles of the TiO_2_-MoS_2_ hybrid at a current density of 100 mA/g.

Sample TM_200_500 (TiO_2_-MoS_2_ hybrid systems hydrothermally treated at 200 °C and additionally calcined at 500 °C) was selected for electrochemical tests, because it is characterized by the best developed crystalline structure. Figure 10a displays the rate performance at various densities. At a current density of 500 mA/g, the capacity of the TiO_2_-MoS_2_ hybrid is 580 mAh/g. When the current density returns to 50 mA/g, the TiO_2_-MoS_2_ material still delivers a capacity of 685 mAh/g.

**Figure 11:**
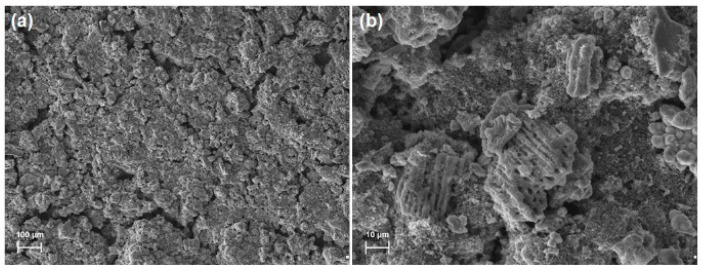
SEM electrodes before (**a**) and after (**b**) the charging/discharging process.

Figure 10b illustrates that the reversible capacity for TiO_2_-MoS_2_ material was 390 mAh/g after 100 cycles at a current density of 100 mA/g. After the second cycle, the TiO_2_-MoS_2_ hybrid material showed a Coulombic efficiency of around 98%. The scanning electron microscopy images for the electrode before and after the charging/discharging process are presented in Figure 11. In the presented SEM images, many spherical particles are observed on the surface of an electrode before the charging/discharging process. Meanwhile, the MoS_2_ nanostructures are difficult to determine. They are most likely related to the presence of Acetylene Black and a binder in the electrode structure. It has been observed that the initial capacity of the TiO_2_-MoS_2_ hybrid is lower than that of pure MoS_2_ (this is due to the presence of TiO_2_ in the electrode structure). However, it should be noted that the cycle stability is superior to that of pure MoS_2_.

Figure 10c shows the voltage profiles of the TiO_2_-MoS_2_ hybrid material during the first, second and 40th cycles at a current density of 100 mA/g at room temperature. According to the previous literature, it was confirmed that, during the first discharge cycle, two voltage plateaus can be observed—about 1.0 V and 0.50 V, respectively. The first voltage plateau is attributed to the Li insertion reaction (it most likely corresponds to the formation of Li_x_MoS_2_). The second one—at 0.50 V—is associated with the reduction process. MoS_2_ is reduced to Mo particles embedded in the LiS_2_ matrix. It was observed that, for the first cycle, the discharge and charging capacities are 680 mAh/g and 610 mAh/g, respectively, which corresponds to a Coulombic efficiency of 89%. The charge and discharge capacities in the second cycle are 622 mAh/g and 608 mAh/g, respectively, which gives a Coulombic efficiency of 98%. The loss of capacity is associated with irreversible reactions that occur during the discharge/charge processes [72,89].

High currents in both the loading and unloading process (in cycles) are used to improve the Coulombic efficiency. However, it should be remembered that high currents do not always lead to good energy efficiency. In addition, heterogeneous precipitation in the form of low solubility (sulfur in the charge cycle and sulfide/disulfide in the discharge cycle) leads to an insufficient use of active substances. Moreover, it should be noted that an insufficient current in the charging/discharging process can lead to failure or insufficient cell capacity throughout the entire process. The observed sudden decrease in the capacity of the high-voltage plateau is associated with the high reactivity of higher-order polysulfides with lithium electrodes.

Very often, we see a rapid decrease in the capacity of Li-S batteries. This phenomenon is explained by the high mobility of the polysulfide form in liquid electrolytes. Damage to the electrode structure by the subsequent precipitation of Li_2_S on the surfaces of both electrodes is associated with a loss of capacity on the low-voltage plateau [90,91,92,93,94]. Li_2_S is formed on the cathode by electrochemical reduction and, on the anode, Li_2_S is formed as a result of the chemical reduction in polysulfides that diffuse from the cathode. In addition, it is easy to observe the passivation layer in the form of a solid Li_2_S layer on the cathode surface. The Li_2_S passivation layer still remains on the carbon-cathode matrix, especially when the cell is fully charged [90]. Not only is this layer responsible for the electrode polarization, it is also responsible for the loss of capacity and high cell resistance [92,93]. In addition, the cell can be damaged by developing microcracks or cracks as a result of stress created during cyclic operation of the cell. The formation of a passivation layer on the anode side can secure the cyclical operation of lithium metal; however, the cell capacity decreases, and its resistance increases. A similar passivation layer of Li_2_S is formed on the anode surface as a result of the surface reaction of polysulfides with metallic lithium [92,93].

In the case of Li-S batteries with liquid electrolytes, the transfer of polysulfide in systems containing sulfur should be mentioned. This phenomenon is associated with several aspects, namely low Coulombic efficiency, high self-discharge, significant sulfur migration and rapid reduction in battery capacity. The uncontrolled process of precipitation in appropriate structures like Li_2_S_2_ or Li_2_S is associated with the mobility of sulfur forms, which, in turn, is caused by the transfer of polysulfide. All these properties have an influence on the charging/discharging profiles of Li-S cells. However, it should be noted that sulfur migration leads to the self-discharge of the system [93].

Electrochemical impedance spectroscopy is a very important measurement technique that provides information based on the study of electrochemical properties. The impedance frequency spectrum contains important information on the electrochemical properties of the studied system (diffusion, charge transfer, electrolyte resistance).

Slight differences were observed when adjusting the replacement circuit. It is worth noting that there are other systems that also fulfill their properties in analyzing impedance spectra. Ivanishchev with co-authors [95,96,97,98,99] give such examples when the electrode surface was calculated. In the abovementioned works, the authors examine cathode behavior in this way, e.g., Li_3_V_2_(PO_4_)_3_ [100]. The circuit they propose involves the transfer in the surface layer of the relative resin of the intercalation material and its mass. To model such a mechanism, two Warburg diffusion impedances connected in series are used, the effect of which is manifested in high and low frequencies [100]. Both these parameters allow for the determination of the diffusion coefficient of lithium ions in the intercalation material (this is used especially when there is a spectrum analysis after the charging/ discharging process).

**Figure 12:**
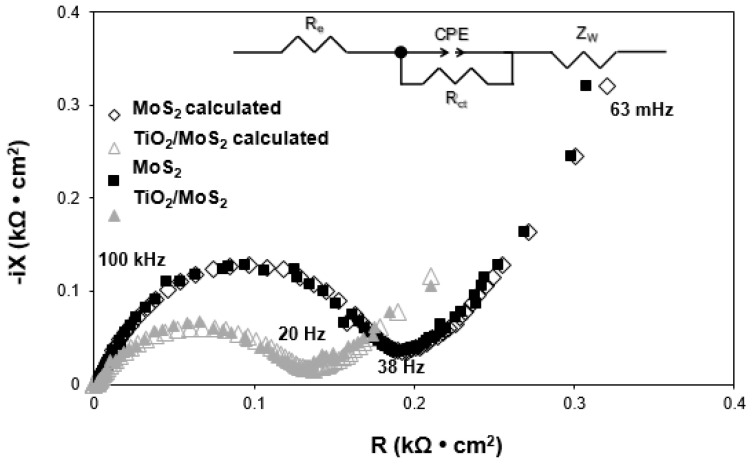
Nyquist plots of the TiO_2_-MoS_2_ and MoS_2_ electrodes (initial spectra and the calculated spectra in accordance with the equivalent circuit model of the system).

Figure 12 shows the Electrochemical Impedance Spectroscopy (EIS) and equivalent circuit model of the tested system. *R_e_* represents the contribution of the resistance of the electrolyte, the electrode and the passivation layer between them. R*_ct_* and Constant Phase Element (CPE) are associated with charge transfer resistance, and Z_w_ with Warburg impedance. For the TiO_2_-MoS_2_ hybrid, the values of *R_e_* and *R_ct_* are 0.0151 kΩ and 0.132 kΩ, much lower than for MoS_2_ (0.0023 kΩ and 0.182 kΩ). In addition, taking into account the electrode surface, the calculated and experimental Nyquist plots were illustrated. It should be noted that the results almost overlap, which indicates a good replacement circuit selection.

Reddy et al. [101] gave an interesting interpretation of the impedance spectra. The authors, in their interpretation, matched all discharging and charging voltages to equivalent electrical circuits, slightly modified from the circuit. The following parameters were taken into account: total resistance of electrolytes and cell components (R_e_); resistance due to surface coating and transfer charge (R_sf+ct_); capacity due to surface layer and double layer (CPE_sf_ + dl); bulk capacity (CPE_b_); bulk resistance (R_b_); Warburg impedance (W_s_); intercalation capacity (C_int_). It has been shown that, in most cases, the anode material showed similarity plot with only one circle in the high to medium frequency range. The authors used an identical substitute circuit for another anode material (nano-(V_1/2_Sb_1/2_Sn)O_4_). Electrochemical impedance spectroscopy was also used to study the kinetics of the anode electrode [102].

This result further confirms that the introduction of TiO_2_ can significantly improve the conductivity of the TiO_2_-MoS_2_ hybrid electrode and significantly accelerate electron transport during the electrochemical lithium insertion and extraction reaction, which results in a significant improvement in electrochemical efficiency.

## 4. Conclusions

TiO_2_-MoS_2_ hybrid materials were successfully synthesized utilizing a hydrothermal approach and were additionally subjected to a calcination process. Our results show that the temperature of the hydrothermal technique, as well as the use of calcination, significantly affected the morphology and crystalline and textural structure of the final hybrid products. The scanning and transmission electron microscopy images demonstrated that TiO_2_ particles are uniformly distributed on MoS_2_ sheets. Moreover, it was proven that additional calcination treatment leads to TiO_2_-MoS_2_ hybrid materials with higher crystallinity.

The addition of TiO_2_ significantly facilitates the transport of electrons and ions and controls the change in the volume of MoS_2_ during the discharge process. The resulting good capacity may be due to the large surface area of the interface with the electrolyte and the shortened Li-ion insertion distance. The results suggest that the TiO_2_-MoS_2_ hybrid is a promising candidate for an anode material in lithium-ion batteries.

## Figures and Tables

**Table 1:** Lattice parameters and phase composition for synthesized materials based on TiO_2_ and MoS_2_.

Sample			TM_180	TM_180_500	TM_200	TM_200_500
**Lattice parameter**	**anatase**	*a* (Å)	3.8390(1)	3.7977(9)	3.7946(1)	3.7674(9)
		*c* (Å)	9.5863(5)	9.4957(2)	95355(2)	9.4556(2)
	**rutile**	*a* (Å)	-	4.6248(1)	-	4.6336(1)
		*c* (Å)	-	3.1342(2)	-	3.1146(2)
	**MoS_2_**	*a* (Å)	3.1332(1)	3.1104(1)	3.0994(2)	3.0704(1)
		*c* (Å)	13.378(4)	13.265(4)	13.384(1)	13.242(4)
**Composition** **(% wt.)**	**anatase**		61(1)	54(1)	77(1)	52(1)
	**rutile**		-	22(1)	-	20(2)
	**MoS_2_**		39(1)	24(1)	23(2)	28(2)
